# Supplementary material for: The Expression of the Immunoproteasome Subunit PSMB9 Is Related to Distinct Molecular Subtypes of Uterine Leiomyosarcoma
Source: Cancers (Basel). 2022 Oct 13;14(20):5007. doi: 10.3390/cancers14205007 (PMC9600211; doi:10.3390/cancers14205007)
Supplement: Supplementary file 1 [file cancers-14-05007-s001.zip › cancers-1936041-supplementary.pdf]

cancers

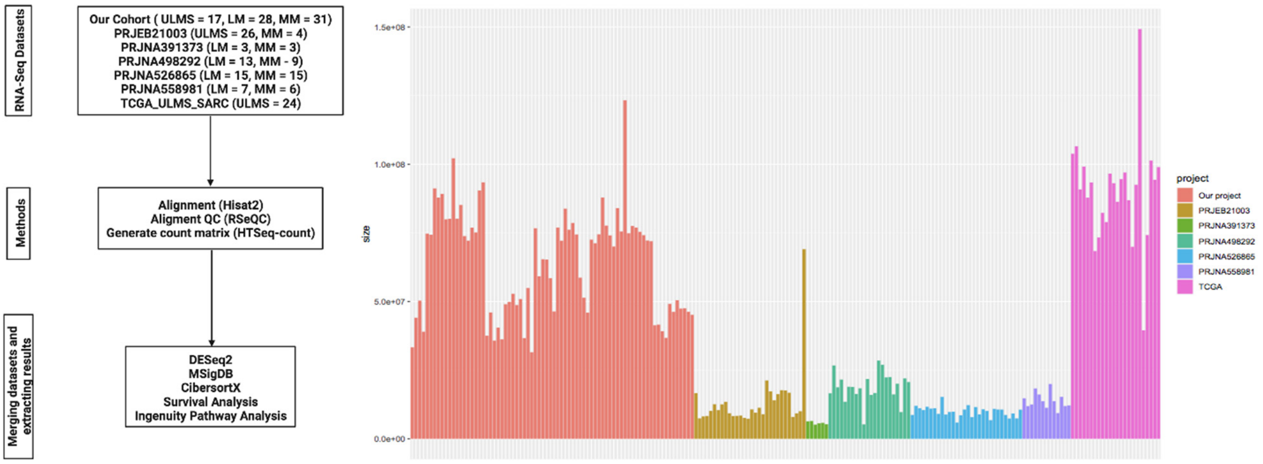

Supplementary Figure S1. (A) Patients samples flowchart; (B) barplot showing different library sizes of each BioProject. TPM normalization can be used in order to integrate the data.

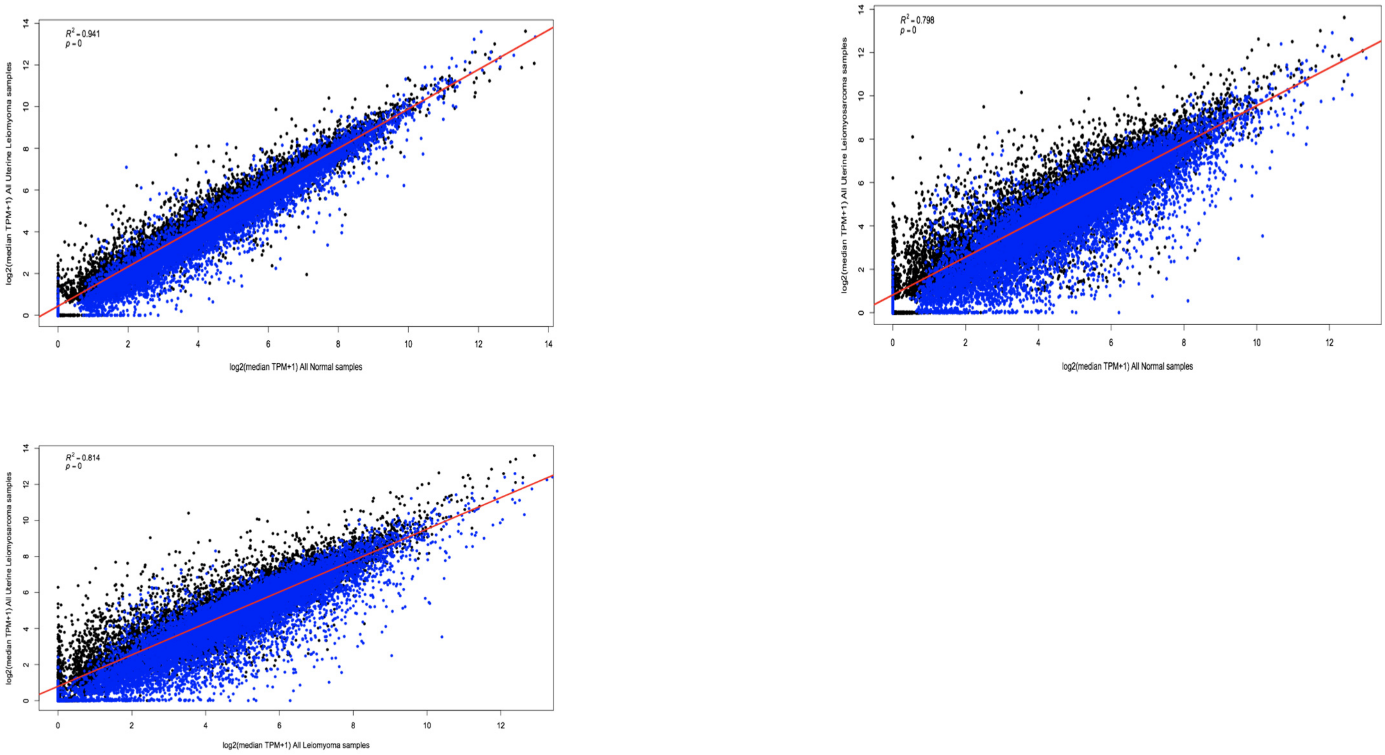

Supplementary Figure S2. (**A**) Concordance of gene expression between normal adjacent myometrium (MM) and leiomyoma (LM) samples after data integration. Each point is a gene, with black points representing protein coding from LM samples and blue points representing protein coding from MM samples. The red line represents the linear regression in the form of Y~X; (**B**) concordance of gene expression between MM and uterine leiomyosarcoma (uLMS) samples after data integration. Each point is a gene, with black points representing protein coding from uLMS samples and blue points representing protein coding from MM samples. The red line represents the linear regression in the form of Y~X; (**C**) concordance of gene expression between LM and uLMS samples after data integration. Each point is a gene, with black points representing protein coding from uterine leiomyosarcoma samples and blue points representing protein coding from leiomyoma samples. The red line represents the linear regression in the form of Y~X.

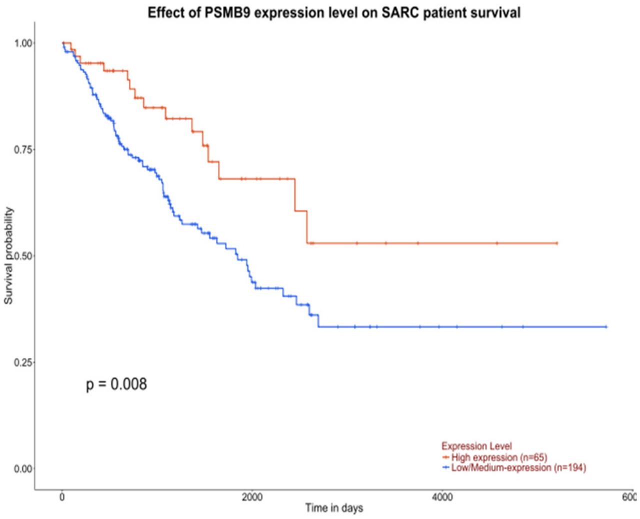

Supplementary Figure S3. Kaplan–Meier curves from all TCGA-SARC samples. High PSMB9 expression group consists of samples with TPM values above upper quartile. Low/medium PSMB9 expression group consists of samples with TPM values below upper quartile.

Supplementary Table S1. The number of samples per group and per project.

Project Name	#Total Samples	#uLMS Samples	#LM Samples	#MM Samples	Project Name
TCGA_uLMS_SARC	24	24	0	0	TCGA_uLMS_SARC
PRJEB21003	30	26	0	4	PRJEB21004
PRJNA526865	30	0	15	15	PRJNA526865
PRJNA498292	22	0	13	9	PRJNA498292
PRJNA558981	13	0	7	6	PRJNA558981
PRJNA391373	6	0	3	3	PRJNA391373
Our project	76	17	28	31	Our project
TOTAL	201	67	66	68	TOTAL

Supplementary Table S2. Analysis of correlation between PSMB9 expression value and CD8+ in the low/high groups. In red, the discordant samples are highlighted.

Sample_name	CD8_value	PSMB9_value	PSMB9_LEVEL_GROUP	CD8_PSMB9_ratio	CD8_PSMB9_LEVEL_GROUP
LMS_P17312_201_S20	0	5.766692075	LOW	0	LOW
LMS_P17312_215_S32	0	0.443936328	LOW	0	LOW
LMS_P17312_237_S52	0	4.872600975	LOW	0	LOW
TCGA.JV.A75J	0	1.311145467	LOW	0	LOW
TCGA.KD.A5QU	0.121089905	6.587302162	LOW	0.018382321	HIGH
TCGA.N1.A6IA	0	5.022424434	LOW	0	LOW
LMS_P17312_203_S22	0	30.15376278	HIGH	0	LOW
LMS_R106	0.383871163	79.08063415	HIGH	0.004854174	HIGH
LMS_R51	0.152514955	32.03993129	HIGH	0.004760152	HIGH
LMS_R59	0.204694647	36.00971655	HIGH	0.005684428	HIGH
TCGA.3B.A9HV	0	26.18430585	HIGH	0	LOW
TCGA.IS.A3K6	0.35202806	54.86086192	HIGH	0.006416743	HIGH
TCGA.JV.A5VE	0.363458438	72.89517077	HIGH	0.004986043	HIGH
